# Supplementary material for: A One Health Approach for Guinea Worm Disease Control: Scope and Opportunities
Source: Trop Med Infect Dis. 2020 Oct 13;5(4):159. doi: 10.3390/tropicalmed5040159 (PMC7709623; doi:10.3390/tropicalmed5040159)
Supplement: Supplementary file 1 [file tropicalmed-05-00159-s001.zip › Table S1.docx]

Table S1. Literature Review Search Strategy.

Project: A One Health approach for Guinea worm disease control: Scope and opportunities

Purpose: Search Syntax | Date: January 2020 | Database: PubMed

| **Set** | **Strategy** | **Results** |
| --- | --- | --- |
| **#1** | Dracunculiases[mesh] or Dracunculosis[mesh] or “Dracunculus medinenses”[mesh] or “Dracunculus medinensis”[mesh] or “Dracunculus Nematode”[mesh] or “Guinea Worm”[mesh] or “Guinea Worm Disease”[mesh] or “Medina Worm”[mesh] or “Dragon worm”[mesh] or Medinensis[mesh] | 902 |
| **#2** | Chad or “Republic of Chad” or Ethiopia or “Federal Democratic Republic of Ethiopia” or “South Sudan” or “Republic of South Sudan” or Angola or “Republic of Angola” or Cameroon or “Republic of Cameroon” or Cameroun or “République du Cameroun” or Mali or “Republic of Mali” | 41,237 |
| **#3** | Dog[mesh] OR "Canis familiaris"[mesh] OR Cat[mesh] OR "Domestic Cat"[mesh] OR "Felis sylvestris catus" OR "Felis domesticus" OR "Felis catus" OR "Felis domestica" OR Baboon[mesh] OR "Savanah Baboon" OR papio OR Frog[mesh] OR rana OR fish OR tilapia OR minnow OR "Mosquitofish" OR "Oreochromis niloticus" OR "Pimephales promelas" OR "Gambusia affinis" | 712,525 |
| **#4** | #1 AND #2 AND #3 | 15 |

Project: A One Health approach for Guinea worm disease control: Scope and opportunities

Purpose: Search Syntax | Date: January 2020 | Database: Web of Science

| **Set** | **Strategy** | **Results** |
| --- | --- | --- |
| **#1** | Dracunculiases or Dracunculosis or “Dracunculus medinenses” or “Dracunculus medinensis” or “Dracunculus Nematode” or “Guinea Worm” or “Guinea Worm Disease” or “Medina Worm” or “Dragon worm” or Medinensis | 356 |
| **#2** | Chad or “Republic of Chad” or Ethiopia or “Federal Democratic Republic of Ethiopia” or “South Sudan” or “Republic of South Sudan” or Angola or “Republic of Angola” or Cameroon or “Republic of Cameroon” or Cameroun or “République du Cameroun” or Mali or “Republic of Mali” | 54,410 |
| **#3** | Dog OR "Canis familiaris" OR Cat OR "Domestic Cat" OR "Felis sylvestris catus" OR "Felis domesticus" OR "Felis catus" OR "Felis domestica" OR Baboon OR "Savanah Baboon" OR papio OR Frog OR rana OR fish OR tilapia OR minnow OR "Mosquitofish" OR "Oreochromis niloticus" OR "Pimephales promelas" OR "Gambusia affinis" | 911,068 |
| **#4** | #1 AND #2 AND #3 | 13 |
